# Developing four cuproptosis-related lncRNAs signature to predict prognosis and immune activity in ovarian cancer

**DOI:** 10.1186/s13048-023-01165-7

**Published:** 2023-04-30

**Authors:** Li Liu, Qing Wang, Jia-Yun Zhou, Bei Zhang

**Affiliations:** 1 Department of Obstetrics and Gynecology, Graduate School of Bengbu Medical College, Bengbu, China; 2 Department of Obstetrics and Gynecology, Xuzhou Central Hospital, Xuzhou, China; 3 Department of Obstetrics and Gynecology, Graduate School of Xuzhou Medical University, Xuzhou, China

**Keywords:** Ovarian cancer, Cuproptosis, Signature, Prognosis, Tumor microenvironment

## Abstract

**Background:**

There has been a recent discovery of a new type of cell death produced by copper-iron ions, called Cuproptosis (copper death). The purpose of this study was to identify LncRNA signatures associated with Cuproptosis in ovarian cancer that could be used as prognostic indicators.

**Methods:**

RNA sequencing (RNA-seq) profiles with clinicopathological data from TCGA database were used to select prognostic CRLs and then constructed prognostic risk model using multivariate regression analysis and LASSO algorithms. An independent dataset from GEO database was used to validate the prognostic performance. Combined with clinical factors, we further constructed a prognostic nomogram. In addition, tumor immune microenvironment, somatic mutation and drug sensitivity were analyzed using ssGSEA, GSVA, ESTIMATE and CIBERSORT algorithms.

**Result:**

A total of 129 CRLs were selected whose expression levels were significantly related to expression levels of 10 cuproptosis-related genes. The univariate Cox regression analysis showed that 12 CRLs were associated with overall survival (OS). Using LASSO algorithms and multivariate regression analysis, we constructed a four-CRLs prognostic signature in the training dataset. Patients in the training dataset could be classified into high- or low-risk subgroups with significantly different OS (log-rank *p* < 0.001). The prognostic performance was confirmed in TCGA-OC cohort (log-rank *p* < 0.001) and an independent GEO cohort (log-rank *p* = 0.023). Multivariate cox regression analysis proved the four-CRLs signature was an independent prognostic factor for OC. Additionally, different risk subtypes showed significantly different levels of immune cells, signal pathways, and drug response.

**Conclusion:**

We established a prognostic signature based on cuproptosis-related lncRNAs for OC patients, which will be of great value in predicting the prognosis patients and may provide a new perspective for research and individualized treatment.

**Supplementary Information:**

The online version contains supplementary material available at 10.1186/s13048-023-01165-7.

## Introduction

Ovarian cancer (OC) is one of the most common malignancies of the genital tract, with high mortality rates and a significant impact on the health of women worldwide [1]. More than 70% of OC patients were diagnosed at an advanced stage due to a lack of clinical characteristics and effective biomarkers during the early stage and therefore missed the opportunity to receive treatment [2, 3]. Although the development of treatment such as targeted therapy and immunotherapy, the 5-year overall survival is still less than 30% due to recurrence, drug resistance and curative uncertainty [4, 5]. Hence, it is imminent to find promising biomarkers for predicting prognosis and develop effective therapeutic strategies.

Programmed cell death (PCD) is an important biological process during tissue homeostasis and animal development [6]. Increasing evidence indicated that PCD such as apoptosis, ferroptosis, autophagy, and others plays vital roles in tumorigenesis, progression as well as metastasis [7–9]. A recent study published in the journal *Science* is first revealing cuproptosis, differs from previous PCDs in its special mechanism that excess intracellular copper induces the aggregation of lipoylated dihydrolipoamide S-acetyltransferase (DLAT), which is related to the mitochondrial tricarboxylic acid (TCA) cycle, ultimately leading cell death [10]. Peter Tsvetkov et al. revealed that copper-induced cell death requires mitochondrial respiration, but ATP from glycolysis has less effect on it. Copper does not directly participate in the electron transport chain (ETC) and only plays a role in the tricarboxylic acid (TCA) cycle. These results suggest a strong relationship between copper-induced cell death and mitochondrial metabolism, and a strong link between copper and the TCA cycle [11]. Although the detailed mechanism underlying the role of cuproptosis in tumors is still unclear, the copper ionophore Elesclomol already helped patients whose tumors depend on mitochondria for energy. This blockbuster study seems to point to a new mode of cell death that has great potential as a target for tumor therapy.

RNAs that are larger than 200 nucleotides, known as long non-coding RNAs (lncRNAs), play an important role in the development and progression of tumors [12, 13]. Recently, a number of studies have revealed that lncRNAs are the crucial mediators in the regulation of PCD in OC. For example, Cai et al. found that *lncRNA ADAMTS9-AS1* inhibit ferroptosis by targeting *microRNA-587/SLC7A11* in OC, which provide a new therapeutic target [14]. The *lncRNA TUG1* induces autophagy-related resistance to paclitaxel in OC via sponging *miR-29b-3p* with *miR-29c6* [15]. In recent years, exploring tumor biomarkers has become an increasingly popular field using bioinformatics analysis in OC research [16, 17]. Nevertheless, the regulation of the lncRNA on the cuproptosis pathway in OC is not known at the present time. A better understanding of the function of CRLs (cuproptosis-related long noncoding RNAs) in OC could lead to a deeper understanding of possible mechanisms and ensure accurate treatment.

To the best of our knowledge, this is the first bioinformatics research integrating lncRNA and cuproptosis in OC to reveal the possible mechanism and find accurate biomarker. Based on the TCGA database, we identified the expression of CRLs in OC patients. Using LASSO-Cox regression analysis can minimize the potential of model overfitting and improve the accuracy of model parameter estimation (including shrinkage and tuning parameters) [18]. Thus, for feature selection and subsequently obtaining an optimal model, we use LASSO-Cox regression analysis for further analysis. By using this analysis, a 4 CRL based risk score (RS) model was constructed. Furthermore, we evaluated the differences in the tumor microenvironment (TME), tumor mutation burden (TMB) and immunotherapy response between two risk groups based on the prognostic model. The relationship between those lncRNAs with OC TME and candidate drugs was further analyzed and functional enrichment analysis was conducted, too. Our studies aim to improve the accuracy of prognostic prediction and elucidate the possible mechanisms of CRL in OC.

## Methods and materials

### Data acquisition and identification of cuproptosis-related lncRNAs

A dataset containing RNA sequence transcriptome data, clinical information, as well as somatic mutation data of patients with OC was obtained from the Cancer Genome Atlas (TCGA) database. (https://cancergenome.nih.gov/). We excluded patients (0/ < 30 OS values) for reducing statistical bias and finally obtained 364 OC patients for subsequent bioinformatics Analysis. After randomization, all OC patients were divided into training dataset (*n* = 184) and testing dataset (*n* = 180). Comparing the clinical characteristics of training and testing datasets was done using Chi-square tests. Besides, We downloaded an independent dataset (GSE138866) from the Gene Expression Omnibus (GEO; https://www.ncbi.nlm.nih.gov/geo/)database to validate the prognostic performance. There are ten Cuproptosis-related genes (*FDX1, LIPT1, DLD, LIAS, DLAT, PDHA1, PDHB, MTF1, GLS,* and *CDKN2A*) that have been identified in previous literature [11]. In addition, CRLs were identified via Pearson correlation following the filter criteria (|Pearson R|> 0.3 and *p* < 0.001).

### Establishment of the CRLs risk model

The clinical characteristics of the training dataset and the testing dataset are shown in Table S1 and the two datasets are consistent in clinical characteristics (*p* > 0.05). We selected CRLs that were associated with OS using univariate Cox regression in the training dataset. Base on the prognostic CRLs, we constructed the risk model using LASSO Cox regression analysis and multivariate Cox regression. The RS was calculated using the following formula:

$$Risk\;score=\sum\nolimits_{k=1}^n{Coef\left(IncRNA\right)\ast expr(IncRNA}^k)$$

The *coef* is the multivariate Cox regression coefficients of CRLs and *expr* is the expression of CRLs. We selected the median RS was the optimal cut-off values and defined the OC patients as two subgroups: low-risk group and high-risk group.

### Assessment the prediction ability of risk model

In this article, we evaluated the prognostic signature internally and externally. The testing and entire datasets from TCGA datasets were used as internal data to assess the prognostic performance of the established risk model. The GSE138866 dataset was regarded as the external data to validate the prognostic performance. We used Kaplan–Meier (K-M) analysis to verify whether the overall survival (OS) of OC samples in the high- and low-risk groups was statistically different using R packages “survival” and “survminer”. The receiver operating characteristic curves (ROC) was used to assess the prediction accuracy. Principal component analysis (PCA) analysis, as the most widely used algorithm for dimensionality reduction of high-dimensional data and model visualization, was used to visualize high-risk and low-risk groups according to the expression of the entire TCGA-OC cohort, 10 cuproptosis associated genes, 129 CRLs, and risk model [19]. The t-distributed stochastic neighbor embedding (t-SNE) analysis also was conducted to test the performance of the established model [20].

### Independent prognostic factor analysis and construction of nomogram

The multivariate Cox proportional hazards regression model was used to evaluate independent association between prognostic signature and patient survival after adjusting for age and grade. Using R package “RMS”, we established a nomogram integrated RS as well as other clinicopathological characteristics (age, grade) to better predict the 1-, 3-, and 5-year OS. Besides, we applied calibration curve analysis to examine the reliability of the established nomogram. Besides, the receiver operating characteristic curves (ROC) and the conformance index (C-index) was used to assess the prediction accuracy.

### Comprehensive analysis of tumor immune microenvironment and somatic mutation

By using R package “ESTIMATE”, we calculate each patient’s immune score, stromal score and estimate score and then observed the difference between high- and low-risk groups. Furthermore, we applied ssGSEA, GSVA, and CIBERSORT algorithms to quantify the infiltration of immune cells and immunological functional enrichment in the tumor immune microenvironment between two subgroups. We applied VarScan software to process the “mask somatic mutation” data from TCGA database [21]. Furthermore, we analyzed the tumor mutation burdens (TMB) in different risk groups using the R package "maftools". We used the median TMB score as a cut-off value to divide patients into high and low TMB groups and then observed the survival difference when combined risk groups.

### Exploration of the immunotherapy response and drug sensitivity

To explore potential therapeutic drugs of OC, we applied the R package “pRRophetic” to calculated the IC50 values of the 138 drugs obtained from the Genomics of Drug Sensitivity in Cancer (GDSC) database. By using Wilcoxon sign rank testing, we compared the IC50 values between high-risk and low-risk groups on the basis of this data. Furthermore, we evaluated the sensitivity of the anti-OC drugs such as (Cisplatin, Paclitaxel, Bleomycin and Gemcitabine) between high- and low-risk groups. The expression levels of four critical immune checkpoint inhibitors (including PD-1, PD-L1, HAVCR2 and CTLA4) were compared between two subgroups to investigate the immunotherapy value of the risk model.

### Functional analysis

Differentially expressed genes (DEGs) were identified using "limma" package between high- and low-risk groups based on the criteria that |logFC|> 1 and adjusted *p*-values < 0.05. GO and KEGG enrichment analyses were applied using the package “cluster Profiler” in R based on the differential expression genes between two subgroups. We performed gene dataset enrichment analysis using GSEA software 4.2.1 (c2.cp.kegg.v7.2.symbols.gmt) (https://www.gsea-msigdb.org/gsea/index.jsp) in order to examine pathways related to high-risk and low-risk groups.

### Statistical analysis

All data analysis and processing based on the platform of R (https://www.r-project.org/) and Strawberry Perl (https://www.perl.org). For the above methods of analysis where no special instructions are given, *p* < 0.05 was considered statistically significant.

## Results

### Identification of CRLs in OC

**Fig. 1:**
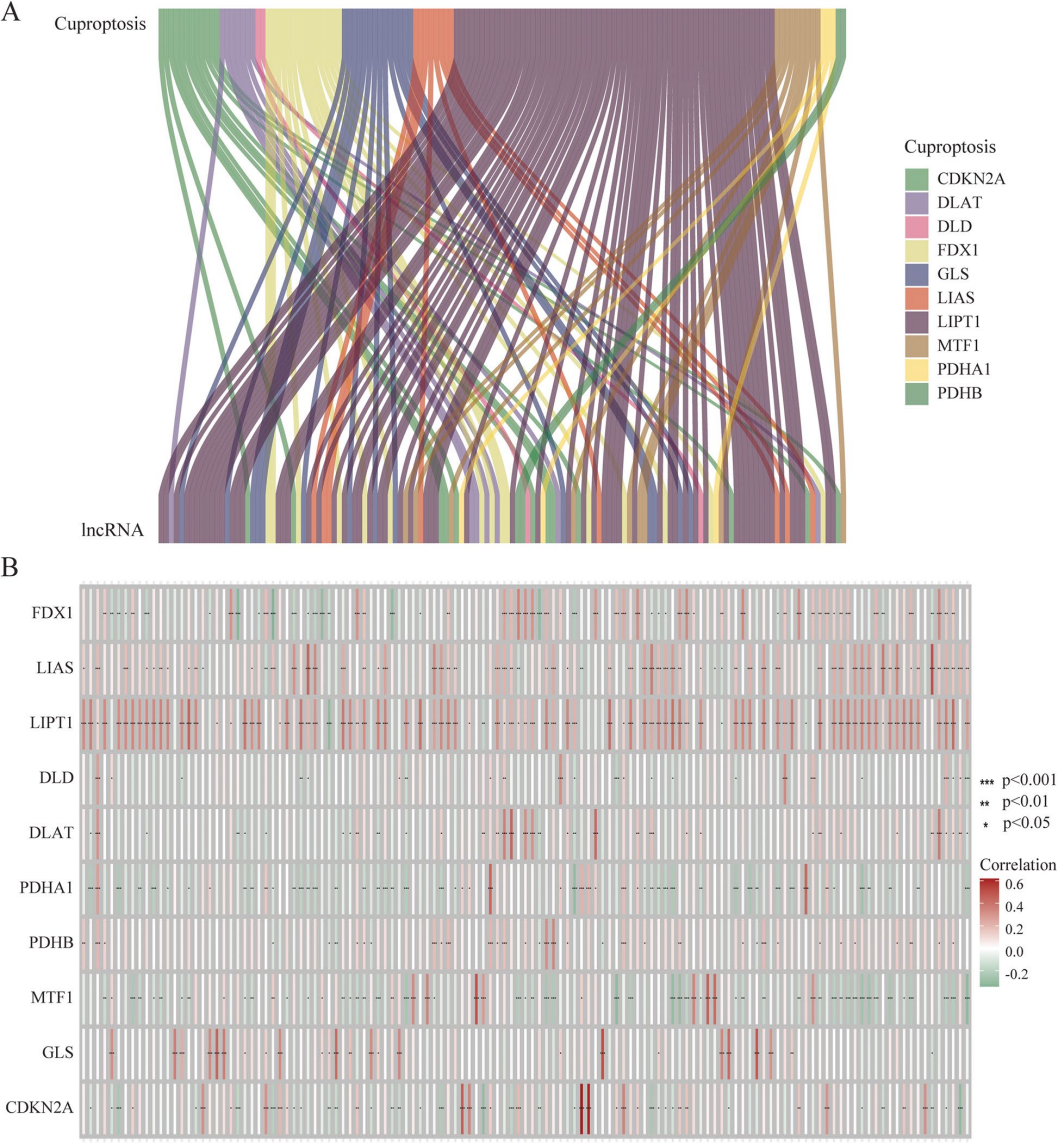
The Sankey diagram and heatmap between 10 CAGs and 129 CRLs. **A** Co-expression network in Sankey diagram for CAGs and corresponding lncRNAs. **B** The heatmap of 10 CAGs and 129 CRLs

Performing Pearson correlation analysis, we identified 129 CRLs whose expression levels were significantly related to expression levels of 10 CAGs (|R|> 0.4 and *p* < 0.001) based on the TCGA OC dataset (Table S2). Using the Sankey diagram and heatmap, we visualized the co-expression network between 10 CAGs and 129 CRLs (Fig. 1A and B).

### Construction and validation of the CRLs-related risk model in training dataset

**Fig. 2:**
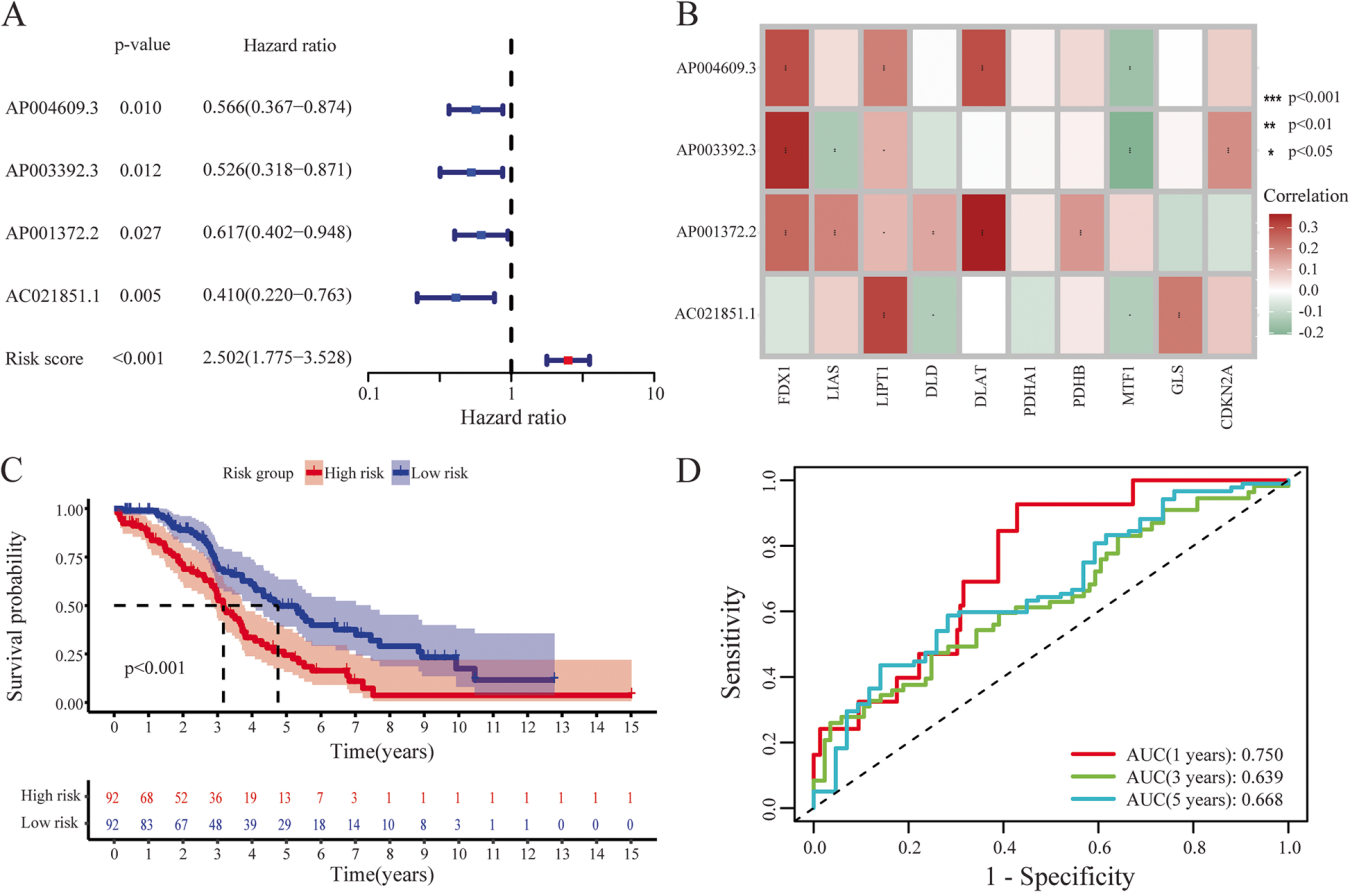
Construction and validation of the CRL risk model based on the training dataset. **A** Forest plot of 4 CRLs within risk model. **B** Heatmap of the correlation between hub lncRNAs and CAGs. **C** Kaplan–Meier curve of high-risk and low-risk patients in the training dataset. **D** The 1-, 3-, and 5-year ROC curves of the training dataset

Twelve CRLs with prognostic significance for OC were identified by a univariate Cox regression analysis in training dataset (Table S3). We performed LASSO regression analysis based on these CRLs to prevent over-fitting of the model, and the prediction accuracy was estimated through 1000 cross validations. We further identified four key CRLs using multivariate Cox regression analysis (Fig. 2A). The heatmap showed the significant expression association between 4 CRLs and 10 CAGs (Fig. 2B). These four CRLs (including *AP004609.3*, *AP003392.3*, *AP001372.2* and *AC021851.1*) were used in the risk model construction and the corresponding coefficients were also given (Table S4). We calculated RS with the formula: RS = expression of *AP004609.3* × (-0.377155976) + expression of *AP003392.3* × coefficient (-0.435241846) + expression of *AP001372.2* × coefficient (-0.590948585) + expression of *AC021851.1* × coefficient (-0.934876246). Then, using the median RS as a cut-off point, patients were divided into high-risk and low-risk groups. We applied the K-M curve and log-rank analysis to test whether there is a significant difference in survival rates between groups with high- and low-risk** (**log-rank *p* < 0.001, Fig. 2C). According to the study results, OC patients in low-risk groups had a higher overall survival rate than those in high-risk groups. The AUC of receiver operating characteristic (ROC) at 1, 3 and 5 years was 0.750, 0.639 and 0.668, indicating the reliability of the model **(**Fig. 2D).

### Validation of the CRLs-related risk model in TCGA and GEO database

**Fig. 3:**
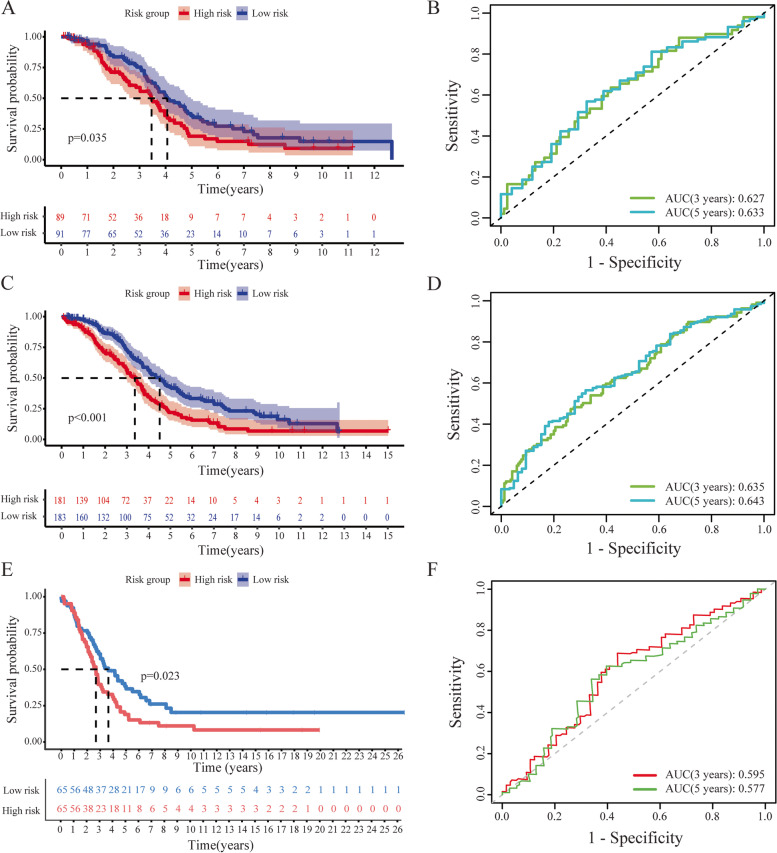
Internally and externally validation separately of the CRLs-related risk model. Kaplan–Meier curves in the testing dataset (**A**), the entire dataset (**C**) and GSE138866 dataset (**E**). The 3- and 5-year ROC curves in the training dataset (**B**), the entire dataset (**D**) and GSE138866 dataset (**F**)

We evaluated the prognostic performance of the risk model by both internal and external validation. As shown in Figs. 3A and C, K-Msurvival curves indicated that patients in high-risk group suffered worse survival than those in low-risk group based on the testing dataset and the entire TCGA-OC dataset, respectively (*p* < 0.05). Besides, the 3- and 5-year AUC for the testing dataset was 0.627 and 0.633 (Fig. 3B), but for the entire TCGA-OC dataset were 0.635 and 0.643 (Fig. 3D). Besides, we tested the prognostic performance in an independent dataset (GSE138866). The results showed that patients in high-risk group had a shorter OS than those in low-risk group (log-rank *p* = 0.023, Fig. 3E). Furthermore, the 3- and 5-year AUC for the GSE138866 database was 0.595 and 0.577, respectively (Fig. 3F). Overall, both internal and external validations indicated that the stability and credibility of our established signature based on CRLs in OC.

### Validation of the CRLs-related risk model by PCA and t-SNE

**Fig. 4:**
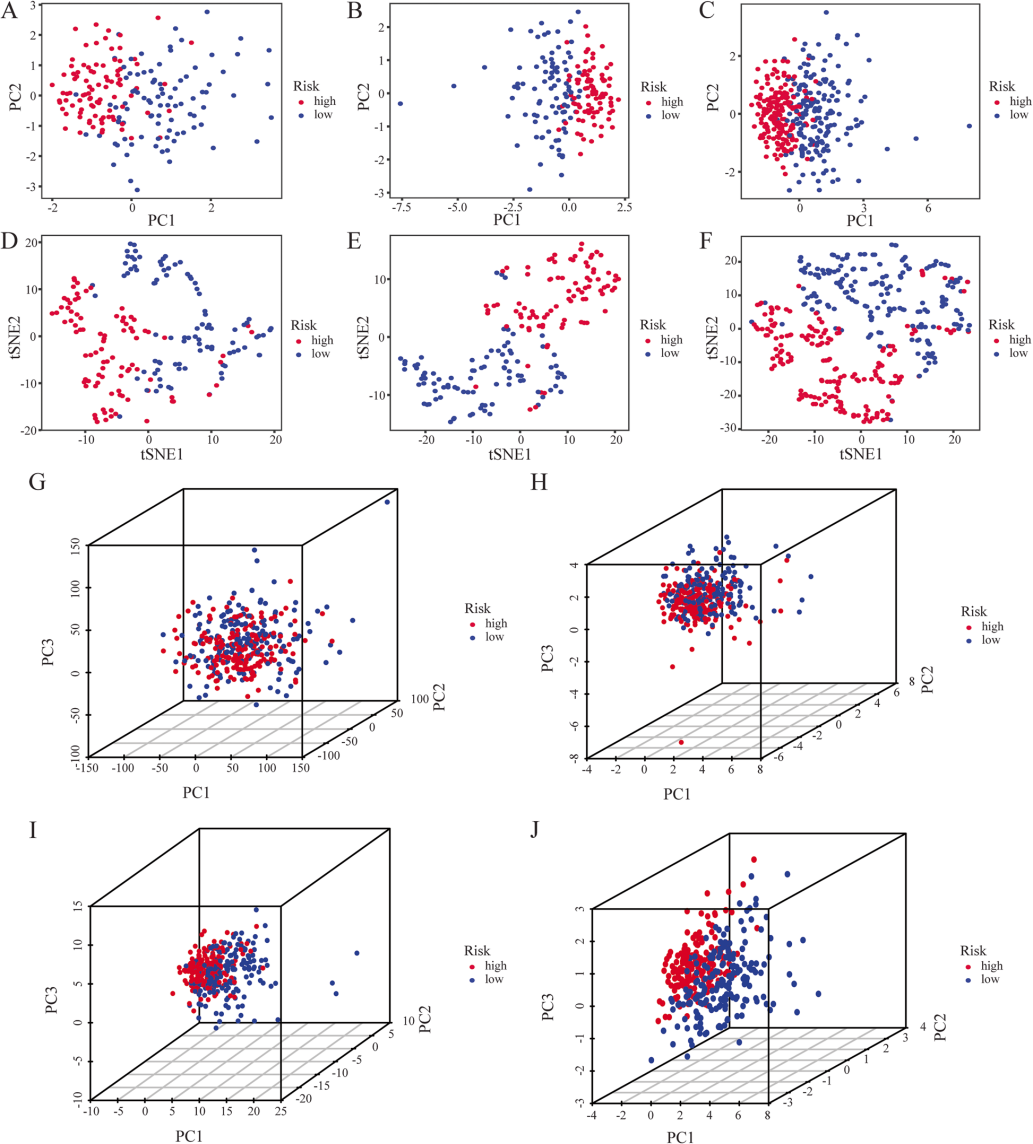
The classification ability of the risk model was evaluated by PCA and t-SNE. PCA map based on training dataset (**A**), testing dataset (**B**), and entire dataset (**C**). t-SNE map based on training dataset (**D**), testing dataset (**E**), and entire dataset (**F**). 3D-PCA map based on entire gene sequencing data of TCGA-OC dataset (**G**), 10 CAGs (**H**), 129 CRLs (**I**), and CRLs-related risk model (**J**)

To assess the classification ability of the risk model based on the 4 CRLs, we firstly applied PCA and t-SNE analyses to the training dataset (Fig. 4A, D), testing dataset (Fig. 4B, E), and entire dataset (Fig. 4C, F). According to the results, the distributions of the high- and low-risk parts were distinct, suggesting the prognostic signature can differentiate between them accurately. The 3D-PCA showed that the distribution of high- and low-risk patients is indistinguishable based on the entire gene sequencing data and CRG expression sets (Fig. 4G, H). There was a tendency of the two subgroups to fall into two subgroups based on CRLs expression set (Fig. 4I). It was, however, the distribution with two subgroups based on the 4 CRLs that appeared to be the most significant (Fig. 4J). These results showed that the risk model had robust and superior classification performance, and the 4 CRLs within the risk model could well reflect the differences between high- and low-risk samples.

### Construction and verification of prognostic nomogram for OC patients

**Fig. 5:**
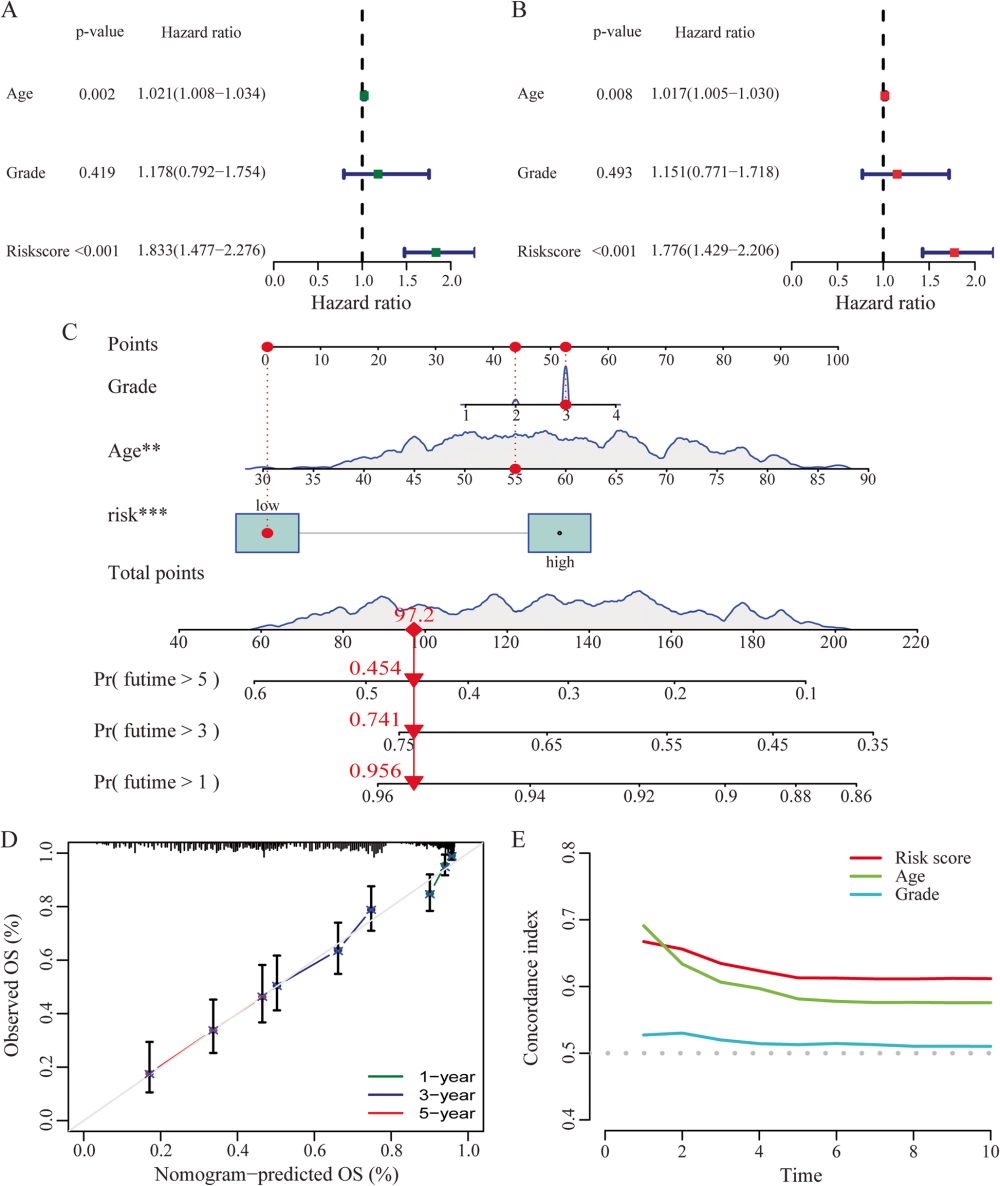
Construction of nomogram and assessment of prognostic performance. Forrest plot of the univariate Cox regression analysis (**A**) and multivariate Cox regression analysis (**B**) in the entire TCGA-OC dataset. **C** The prognostic nomogram constructed based on the RS of CRLs and clinicopathological parameters including age and grade. **D** The calibration curve of the nomogram. **E** C-index of the RS and clinical characteristics

We performed the univariate and multivariate Cox regression analysis to test the prognostic correlations. The results showed that the RS calculated by four-CRLs signature was significantly associated with OS using univariate Cox regression analysis (Fig. 5A) and was an independent prognostic factor after adjusting for age and grade using multivariate Cox regression analysis (Fig. 5B) in the entire TCGA-OC dataset. By combining four CRLs features, we constructed a prognostic nomogram to predict the possibility of 1-year, 3-year and 5-year OS. As shown in Fig. 5C, the score assigned to each factor is proportional to its risk contribution to survival. We verified the accuracy of the nomogram using calibration curves, and found a high degree of accuracy between actual values and predictions (Fig. 5D). Besides, the C-index of RS was higher than that of other predictors, suggesting that the risk model has a significant advantage in predicting the OS of OC patients (Fig. 5E).

### Association of risk model with somatic mutation landscapes and TMB

**Fig. 6:**
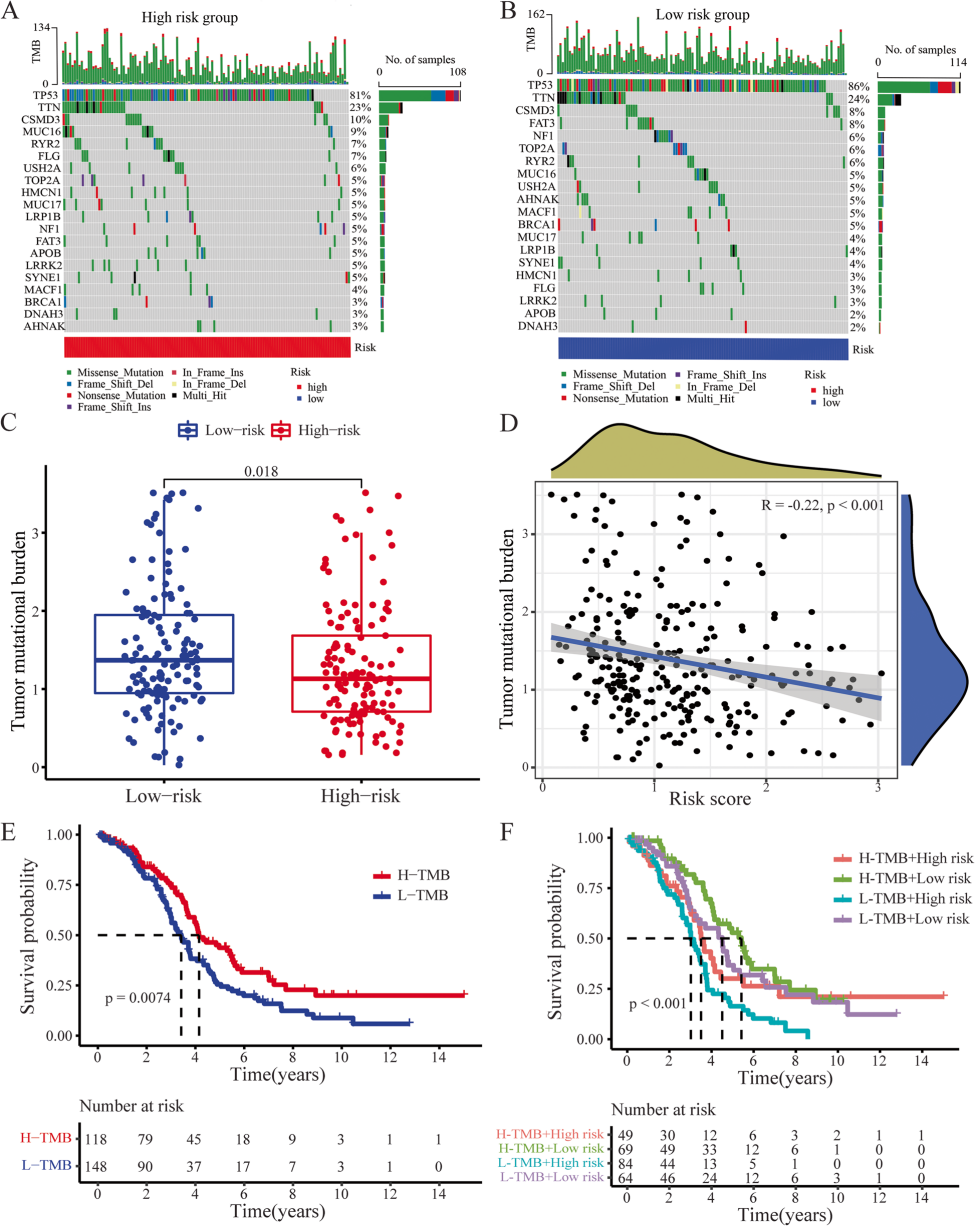
The association between the risk model and the somatic mutation landscapes and the TMB. Mutation information of the genes with high mutation frequencies displayed by waterfall plot in the high-risk group (**A**) and low-risk group (**B**). **C** The TMB differences between high- and low-risk groups. **D** Correlations between RS and TMB. **E** Kaplan-Meier curve between the high- and low-TMB groups. **F** Kaplan-Meier curves of OS for patients with OC and different TMBs and risk scores

It has been found that TMB is positively correlated with tumor stage, grade, and immune infiltrating cells according to research [22]. Next, we analyzed and compared the mutation information between the high- and low-risk groups. According to TCGA somatic mutation data, the mutation status of the top 20 genes with the highest change frequency are shown in Fig. 6A and B for high- and low-risk groups, respectively. The results showed that mutation rate was different between high- and low-risk groups, such as TP53, MUC16 and FLG. Furthermore, TMB scores in the low-risk group were higher than those in the high-risk group (*p* = 0.018, Fig. 6C). It was found that there was a negative correlation between the TMB and the risk model (Fig. 6D) (R = -0.22, *p* < 0.001). Besides, K-M analysis showed that patients in the high-TMB group had a significantly better OS than patients in the low-TMB group (*p* = 0.0074, Fig. 6E). We further found that patients in high-risk group with low-TMB suffered worse survival than those in low-risk group with high-TMB (Fig. 6F).

### Comprehensive immune-infiltration analysis based on risk model subgroups

**Fig. 7:**
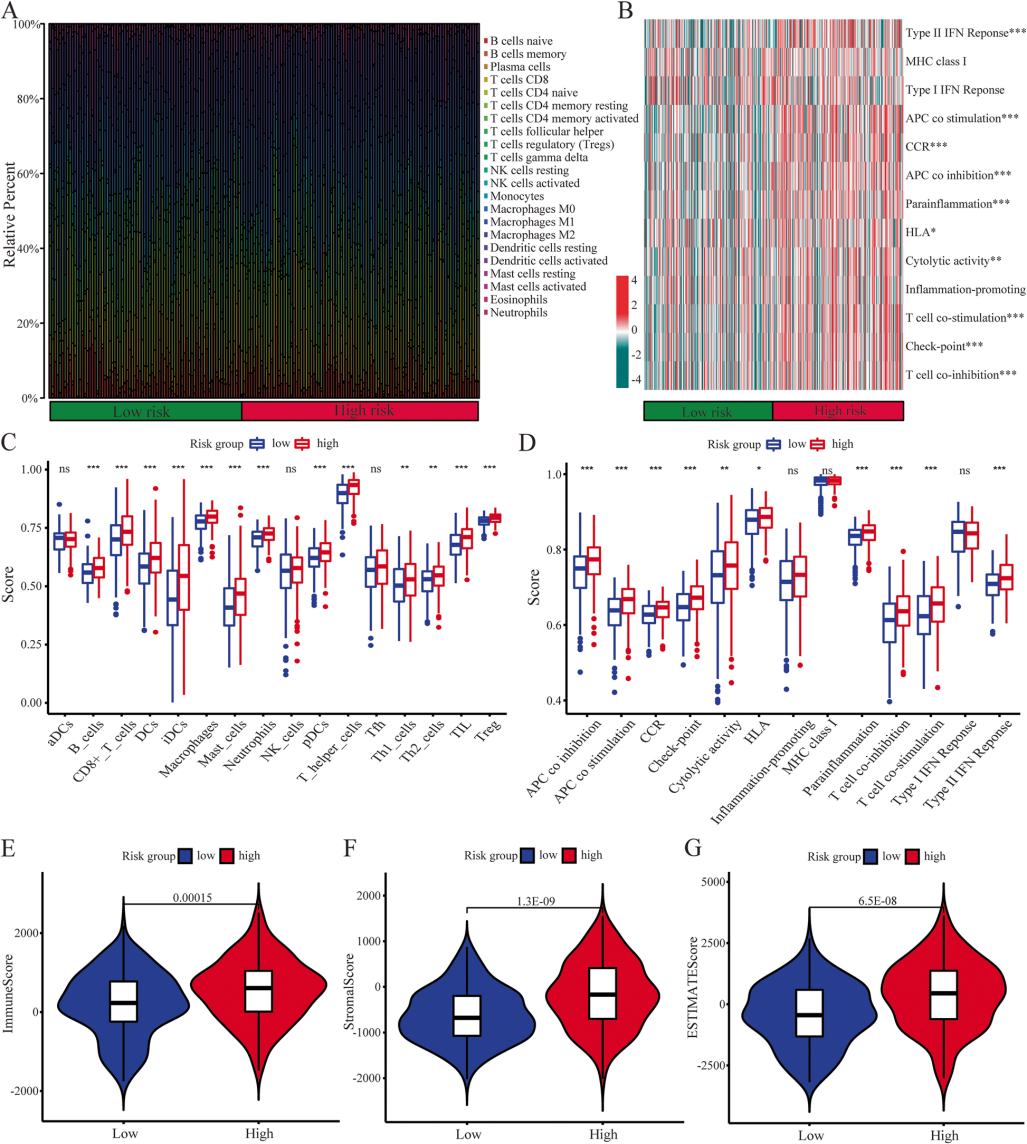
Analysis of immune-infiltration in different risk group. **A** Box plot of the proportion of 22 immune cell components. **B** The GSVA of immune-related pathways between two groups. The difference of immune cell infiltrations (**C**) and immune functions (**D**) between high- and low-risk groups. The differences of the immune score (**E**), stromal score (**F**), and estimate score (**G**) between high- and low-risk groups, respectively. *p < 0.05, **p < 0.01, and ***p < 0.001; ns, not significant

The proportion of 22 immune cell infiltration calculated by CIBERSORT algorithm were compared between high- and low-risk groups. As shown in Table S5, Fig. 7A, there was a significant difference between risk subgroups with respect to specific immune cells, like Macrophages, T cells, resting NK cells, and so on. Besides, the GSVA enrichment analysis revealed that OC patients in the high-risk group were significantly related to immune pathways and functions such as Type II IFN Response, CCR, APC co inhibition, Para inflammation, T cell co-stimulation, T cell co-inhibition and Check-point (Table S6, Fig. 7B). We further conducted the ssGSEA algorithm to explore the difference of immune cell infiltration and immune response for OC patients between high- and low-risk groups (Table S7). The results of immune cell infiltration suggested that infiltration proportions of B cells, CD8 T cells, DCs, Macrophages, Neutrophils, Treg, and T helper cells were obviously increased in the high-risk group (Fig. 7C). As shown in Fig. 7D, immunological function shows significant differences between low- and high-risk groups for all immunological functions except inflammation-promoting, MHC class I, and Type I IFN response (*p* > 0.05). Furthermore, we calculated immune score, stromal score, and estimate score for each OC patient in TCGA-OC dataset using the ESTIMATE algorithm. According to Fig. 7E-G, OC patients in the high-risk group had significantly higher immune, stromal and estimate scores.

### Prediction of the clinical treatment and drug sensitivity analysis based on the risk model

**Fig. 8:**
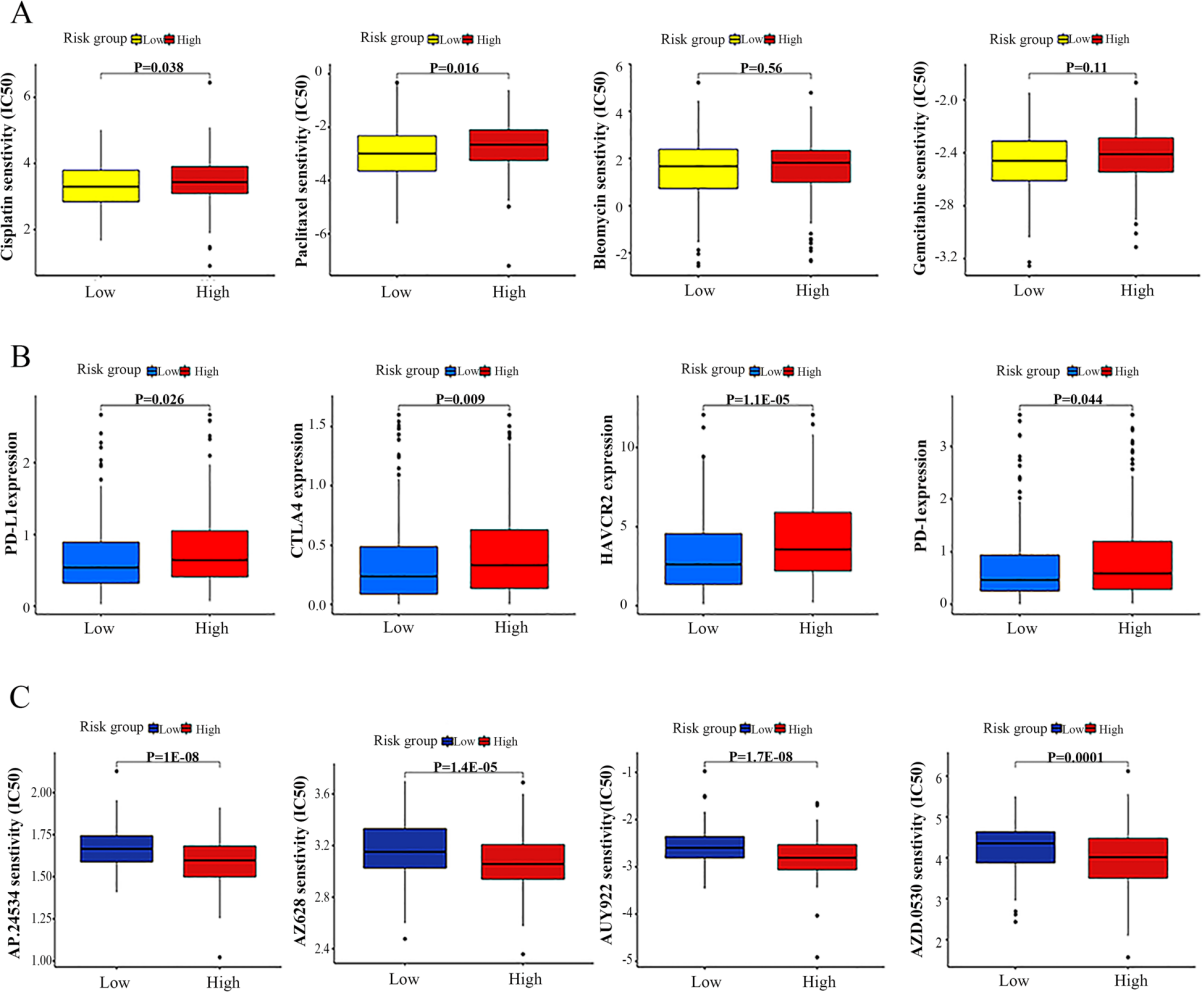
Association of the risk model with chemotherapy and analysis of potential drugs. **A** Evaluation of IC50 for four common chemotherapeutic medicines. **B** Expression levels of PD-L1, CTLA4, HAVCR2, PD-1 in the high- and low-risk groups. **C** Differential analysis of potential drug sensitivity

In light of the significantly different prognosis of OC patients in two subgroups, we decided to further screen potential drugs in order to better achieve targeted therapy. The IC50 values of four common chemotherapeutic medicines were quantified in OC patients, and two were statistically different between risk groups. In detail, the IC50 levels for cisplatin and paclitaxel were significantly higher in the high-risk group of OC patients (Fig. 8A, *p* < 0.05), which indicates that the low-risk group was more sensitive to the above chemical drugs. For Bleomycin and Gemcitabine, no statistical differences in drug sensitivity were observed between two groups. Furthermore, we investigated how two subgroups of patients expressed ICI-related biomarkers. It was noticed that high risk group patients showed high expression of PD1, CTLA4, PD-L1 and HAVCR2, suggesting they might benefit from the above immune therapy (Fig. 8B, *p* < 0.05). As a result of the potential drugs analysis, we found that 4 potential drugs (AP.24534, AZ628, AUY922, and AZD.0530) had significantly higher IC50 values in the low-risk group, suggesting that these drugs may be more suitable for patients in high-risk groups (Fig. 8C, *p* < 0.001).

### Exploring the potential mechanism based on the degs between two subgroups

**Fig. 9:**
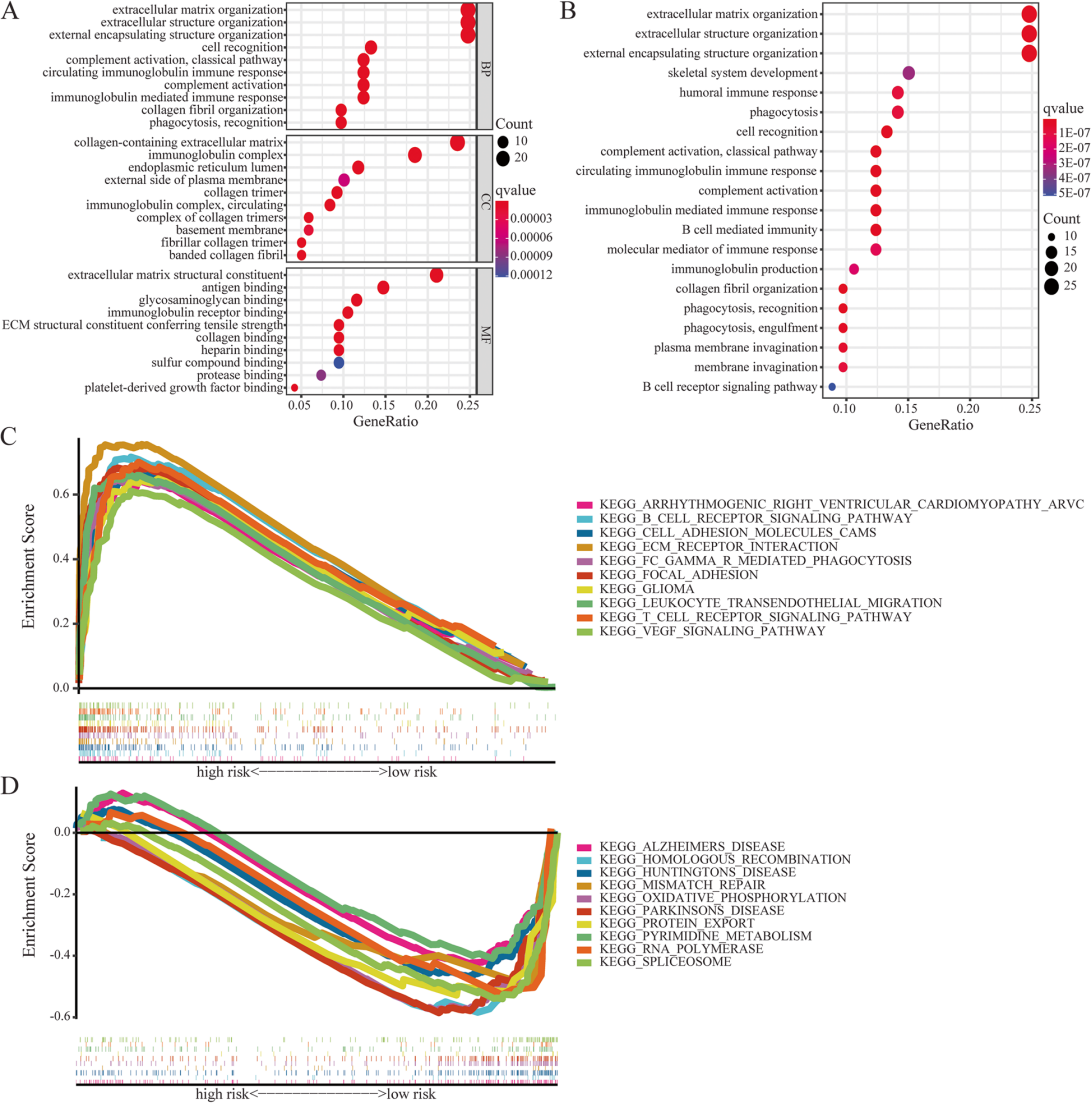
Exploring the potential mechanism based on the DEGs between on two subgroups. **A** GO analysis based on DEGs between high- and low-risk groups. **B** KEGG analysis based on DEGs between high- and low-risk groups. The top 10 pathways significantly enriched in the high-risk group (**C**) and low-risk group (**D**) using gene set enrichment analysis respectively

In order to investigate the potential biological functions and signal pathway with 4 CRLs within the risk model, we identified DEGs between high- and low-risk groups for enrichment analysis. A total of 163 DEGs were selected between two subgroups (Table S8). Based on these DEGs, we performed GO terms and KEGG pathway analysis to explore the underlying molecular mechanism (Table S9). As shown in Fig. 9A, DEGs were significantly enriched in immune-related biological processes, including extracellular matrix, extracellular structure and external encapsulating structure organization based on GO enrichment. KEGG enrichment analysis presented that these DEGs were primarily connected with extracellular matrix organization, extracellular structure organization and external encapsulating structure organization **(**Fig. 9B). By using GSEA software, we were able to explore further differences between high-risk and low-risk groups in terms of biological functions (Fig. 9C, D, Table S10). Pathways such as ECM receptor interaction, T and B cell receptor signaling pathway were significantly enriched in the high-risk group, while pathways like homologous recombination RNA polymerase were highly enriched in the low-risk group.

## Discussion

Women who are diagnosed with ovarian cancer account for 2.5% of all cancers in women, yet they also account for 5% of all cancer-related deaths in women, due largely to advanced diagnosis [23]. Recent studies have suggested that there may be a completely new form of programmed cell death: copper-dependent program cell death known as cuproptosis [11]. Copper is an essential component of many biochemical reactions and is widely involved in a variety of cellular functions, such as cell metabolism, growth, and proliferation, protein activity regulation, as well as apoptosis, autophagy, and other cellular processes [24, 25]. There could be severe consequences if mutations lead to the overloading of copper. In spite of that, it is feasible to manage intracellular copper levels within a certain range in order to selectively kill tumor cells [26]. As a result, cuproptosis has the opportunity to play a role in the treatment of cancer. Studies conducted recently have shown that Long non-coding RNA (lncRNA) has proven to be a key regulatory factor for multiple cancers [27], including OC [28]. Therefore, based on the above evidence, we established a novel OC predictive risk model from CRLs and explored how these lncRNAs affect OC progression.

Based on our initial findings, we identified 4 lncRNAs with functions related to the regulation of cuproptosis and increased prognostic value, providing theoretical support for further studies. Our surprise was finding that, except for *AP001372.2*, which was studied earlier, all three CRLs were studied for the first time. The research group of Liu et al. have developed a risk model using cuproptosis-related lncRNAs, including *AP001372.2*, to predict the outcome of head and neck squamous cell cancer patients [29]. There was potential for them to become OC prognostic markers, which deserve to be explored and studied further. A risk model for predicting OC patients' OS based on 4 CRLs was then developed and validation. To explore the potentially molecular mechanisms by which 4 CRLs affect OC, we performed an enrichment analysis based on DEGs between high- and low-risk groups. Our study found that in GO analysis and KEGG analysis, the DEGs in the high and low risk groups were highly directed to extracellular matrix-related signaling pathways and biological processes. The components of extracellular matrix (ECM) are extremely complex, and they are responsible for the transmission of information between cells. With the deepening of research, it has been found that changes in the content and arrangement of ECM components are often closely related to the occurrence and development of cancer, and the alienated ECM constitutes an important layer in the survival environment of tumor cells [30, 31]. A recent blockbuster study by Laura S M Lecker et al. pointed out that macrophages can change the ECM components of OC by producing TGFBI, resulting in an immunosuppressive environment that is conducive to tumor growth, which is critical for the proliferation and migration of OC cells [32].

In order to develop an OC, immune regulation is a crucial factor that is widely recognized [33]. Counts and proportions of tumor-invading immune cells play a critical role in cancer development and immunotherapy response [34], as well as patient survival. Study findings suggest there is crosstalk between CRLs and immune cells, according to the CRLs signature we detected. There was a statistically significant difference between high and low risk groups with regard to the Macrophages, T cells (T helper, CD4, CD8, Treg), resting NK cells in specific immune cells. It has been shown that Tregs play a critical role in suppressing antitumor immune responses and are associated with poor survival rates [35]. Our findings of abundant Tregs in the TME of high-risk patients are consistent with this. These results suggest that higher levels of immunosuppression in the TME may result in a poorer prognosis for high-risk patients, and this contributes to tumors progression. It is thought that immune cell infiltration in the TME plays a key role in tumorigenesis and progression, and that it influences a cancer patient's prognosis [36]. As immune cells and stromal cells make up TME, immune and stromal scores were associated with OC clinical characteristics and prognosis. According to the ESTIMATE algorithm, a high-risk group had higher immune and stromal scores than a low-risk group. Considering this, it can be hypothesized that cuproptosis may be associated with the involvement of the TME, thereby regulating the occurrence and development of neoplasms. Further, we found significant differences in TMB and somatic mutations between high- and low-risk groups. As previously reported, we have confirmed that higher TMB is associated with better prognosis in OC patients [37], which is consistent with the findings in this study.

Meanwhile, we also evaluated the sensitivity of high-risk versus low-risk patients to commonly used chemotherapeutic drugs, so that we could better guide clinical treatment. Ultimately, we found that cisplatin, paclitaxel, bleomycin, gemcitabine had higher IC50 levels in the high-risk group than in the low-risk group, indicating that the low-risk group of OC patients was more sensitive to these drugs. Additionally, a number of potential compounds have been screened that might provide some new treatment options. As well, the introduction of immune therapies utilizing checkpoint inhibitors has improved survival rates among OC patients [38]. Therefore, we wanted to make an attempt to explore the expression of immune checkpoint in high- and low-risk groups. Our results showed that the expressions of PD1, CTLA4, PD-L1 and HAVCR2 in high-risk patients were higher than those in low-risk patients.

However, we have to admit that this study has some limitations. A major part of the queue in this study is derived from the TCGA database. Additionally, cuproptosis death mechanism is a newly discovered mechanism of programmed cell death, and the studies related to it are scarce. To further verify the feasibility of the prediction model and its potential mechanism, we will collect more samples and perform experiments.

## Conclusion

In the present study, the first attempt has been made to analyze the mechanism of regulation of lncRNAs on cuproptosis in OC cells. Using the relevant data of OC patients in TCGA, we constructed a lncRNA prognosis prediction model based on the regulation of the cuproptosis process. This model can shed new light on the diagnosis and treatment of OC.

## Supplementary Information

**Additional file 1.**

## Data Availability

The datasets analyzed for this study can be found in the TCGA (https://cancergenome.nih.gov/). Further inquiries can be directed to the corresponding author.
